# A Comparison of Randomizing Either One Eye or Both Eyes in Clinical Trials for Stargardt Disease Type 1

**DOI:** 10.1016/j.xops.2025.101021

**Published:** 2025-11-26

**Authors:** Jeroen A.A.H. Pas, Patty P.A. Dhooge, Catherina H.Z. Li, Rob W.J. Collin, Carel B. Hoyng, Joanna IntHout

**Affiliations:** 1 Department of Ophthalmology, Radboud University Medical Center, Nijmegen, the Netherlands; 2 Donders Institute for Brain, Cognition and Behaviour, Nijmegen, the Netherlands; 3 Department of Human Genetics, Radboud University Medical Center, Nijmegen, the Netherlands; 4 IQ Health Science Department, Radboud University Medical Center, Nijmegen, the Netherlands

**Keywords:** Stargardt disease, Clinical trials, Sample sizes, Endpoints, Fundus autofluorescence

## Abstract

**Objective:**

Designing a clinical trial for rare diseases such as Stargardt disease type 1 is challenging due to the limited patient population. In traditional clinical trial designs for inherited retinal diseases, often only 1 eye of each patient is used as the treated eye or the sham, disregarding half of the available eyes.

This study explores a trial design in which both eyes are included, with the fellow eye serving as the control, maximizing the use of available data and enhancing statistical power.

**Design:**

Retrospective analysis of natural history data to conduct sample size calculations.

**Participants:**

Patients with genetically solved Stargardt disease type 1 who had at least 2 fundus autofluorescence measurements obtained within 5 years of each other. Retrospective data of 164 patients were included for analysis.

**Methods:**

The required sample sizes for 1-eye and paired-eye study designs were calculated using retrospective natural history data on the progression of definitely decreased autofluorescence quantified from fundus autofluorescence imaging.

**Main Outcome Measures:**

Required sample size for a clinical trial.

**Results:**

Sample size calculations showed that 170 patients are needed for a 2-year clinical trial with a 1-eye design, decreasing to 99 patients for a 5-year trial. When using a paired-eye design, 64 patients are needed in a 2-year trial, decreasing to 28 patients in a 5-year trial. When using a paired-eye design and requiring definitely decreased autofluorescence atrophy in both eyes at inclusion, 37 patients were needed in a 2-year trial, decreasing to 16 patients in a 5-year trial.

**Conclusions:**

Using a paired-eye design for a clinical trial in Stargardt disease type 1, with definitely decreased autofluorescence atrophy growth rate as the primary end point, is more efficient than a 1-eye design. Implementing additional inclusion criteria, such as requiring definitely decreased autofluorescence atrophy in both eyes at baseline, further reduces the number of patients needed to achieve sufficient statistical power. This approach enhances the feasibility for trials in Stargardt disease type 1 where patient availability is limited.

**Financial Disclosure(s):**

Proprietary or commercial disclosure may be found in the Footnotes and Disclosures at the end of this article.

Therapeutic options for inherited retinal diseases (IRDs) such as Stargardt disease type 1 (STGD1) are upcoming, yet clinical trials face numerous challenges that can lead to failure. Early-phase clinical trials need to evaluate therapeutic effects, within a few years of trial duration, and with limited patient numbers.^1^^,^^2^ Stargardt disease type 1 is a rare disease with an estimated prevalence of 1:10 000 to 8000.^3^ Strict eligibility criteria limit the number of eligible patients for a trial. This small sample size limits the power of a clinical trial to detect treatment effects.

To address this issue, we may need to consider moving away from the traditional 1-eye trial design where only 1 eye per patient is randomized for treatment. Fifty-two percent of the clinical trials published in top-tier ophthalmic journals between January 2020 and December 2021 had used a 1-eye design and only 10% had used a paired-eye design.^4^ In paired-eye designs, 1 eye of each patient is treated, whereas the other eye serves as control.^4^^,^^5^ To get a valid estimate of the treatment effect, this type of trial design can only be used with locally administered therapies that do not affect the fellow eye. A substantial part of the currently emerging treatment options consists of such local therapies, especially genetic therapies, which are administered by intravitreal injection in the case of antisense oligonucleotide therapy and by subretinal injection in the case of adeno-associated virus-mediated gene therapy.^6^, ^7^, ^8^

When using a paired-eye design with the fellow eye as control, an end point with consistent progression over time is the most preferable, to ensure (if not treated) a similar progression in both eyes as much as possible. In STGD1, one of the most-used trial endpoints that shows linear progression (after square root transformation) is atrophy progression measured on fundus autofluorescence (FAF). This outcome reflects the disease progression and can be measured at each eye separately, and thus can be used for evaluation of the treatment effect (Pas, 2024, unpublished data). However, despite the linear progression, FAF shows some asymmetric intereye progression.

By using natural history data from a large cohort of STGD1 patients with FAF measurements, this study aims to explore the possibility of randomizing eyes, using the fellow eye of a STGD1 patient as control instead of using the eye of a different patient.

## Methods

Participants were included from the Radboudumc STGD1 database, which contained natural history data of 421 patients with genetically solved (2x *ABCA4*) STGD1 at the time of inclusion. We included patients with at least 2 FAF images from separate visits, only including visits within 5 years of the most recent visit of a patient. Visits more than 5 years before the most recent visit were excluded. Visits with atrophy crossing the border of the FAF image were excluded as well. Statistical analyses were performed using the statistical software R version 4.1.3^9^ in combination with RStudio version 2022.02.1 (Posit Software, PBC). The Institutional Ethics Committee, CMO Oost-Nederland, ruled that ethical approval was not required for this study and waived the requirement for informed consent (file no. 2022-15718). The study was performed in adherence to the tenets of the Declaration of Helsinki.

### Data Collection

Selection of the FAF images and grading of the DDAF (definitely decreased autofluorescence) area on FAF imaging was performed for an earlier study,^10^ using the Heidelberg Regionfinder (Heidelberg Engineering) semiautomatic grading software. No changes to the measured values were applied.

In the selected natural history data, the time at which the most recent FAF image of every patient was taken, was defined as time = 0, and times of the earlier captured FAF images were defined as negative timepoints, for example, –3.4 years. The variation at timepoint 0 reflects the variation that can be expected at the baseline visit in a future trial, in which patients will be included regardless of their disease duration.

### Modeled Clinical Trial Designs

Three different clinical trial designs were defined. The first design resembles the current clinical practice, in which a 1:1 randomization is applied to the patients and only 1 eye per patient is used, the “study eye” of a patient: the 1-eye design. The study eye is here defined as 1 randomly selected eye per patient, receiving either treatment or sham. The contralateral eye will be ignored in this design. The second design involves randomization across both eyes of each patient: the paired-eye design. In this design, the randomly chosen study eye will receive treatment, whereas the fellow eye of the patient will receive a sham treatment. The third design is an advanced version of the second design, again randomizing across both eyes of a patient, but with an additional inclusion criterion stating that both eyes of the patient must have DDAF atrophy at the start of the clinical trial, to ensure measurable DDAF atrophy progression in each eye during the trial: the paired-eye design with additional inclusion criterion. As atrophy progresses over the years, the difference between the treated and control eye is expected to increase over time. For each design, sample sizes were calculated for clinical trials with durations of 2, 3, 4, and 5 years. We chose an upper limit of 5 years for the clinical trial duration because in our opinion it will not be economically feasible to conduct longer clinical trials.

### Calculating the Natural Atrophy Progression

The primary end point in the clinical trial will be the square root of DDAF progression. Therefore, for the sample size calculations, we need the mean slope of natural $\sqrt{\text{DDAF}}$ progression, together with its standard deviation (SD) and the correlation between the progression in the left and right eye, the intraclass correlation coefficient (ICC). Using all 5-year data for robust estimates of the progression: the mean slope of $\sqrt{\text{DDAF}}$ progression was calculated per eye and patient. Subsequently, for each trial duration, a separate SD of the slopes of $\sqrt{\text{DDAF}}$ progression was calculated as the average of the SDs of the right and the left eye. For a 2-year design, the SD was calculated using data from 2 years before the last visit to last visit, and similarly, for longer durations, the corresponding retrospective time points were used. This approach ensures that each design’s SD reflects the variability in progression relevant to the planned trial duration. On these same data, the ICC of the intereye correlation was calculated using a linear mixed-effects model with only an intercept and a random effect per patient, reflecting the nesting of 2 eyes within 1 patient.

### Effect Size Calculation

Next, effect sizes were calculated for each design. It is assumed that the treatment will have an inhibitory effect on the atrophy progression, but that there may be a delay before it is effective. Further, it is conservatively assumed that there may be contamination, with a corresponding small effect of the treatment on the control eye. The resulting treatment effect is calculated using the following formula:

$$\Delta =\left(mean.\sqrt{DDAF}.slope\times inhibitory.treatment.effect\;\right)\times \left(retained.study.duration\;\right)\times$$

(1–contamination.rate)

This effect size (Δ) represents the expected effect size of the treatment on the slope of $\sqrt{\text{DDAF}}$. We assumed an 80% inhibitory treatment effect by the therapy, which means that the $\sqrt{\text{DDAF}}$ slope of the treated eye will be 20% of the slope of an untreated eye. Although this represents a high level of progression rate inhibition, we choose for this 80% inhibition because we find it clinically relevant, as it slows the process by a factor of 5.

Subsequently, various penalties were implemented to counter a too-optimistic value of the effect size. First, a penalty of a 3-month study duration delay was applied to all designs because it is not clear whether the applied therapy will work immediately. The retained study duration in this formula resembles the resulting study duration: For example, in a 2-year clinical study, a 3-month delay will lead to 24–3 = 21 months of effective therapy. In that case, the retained study duration will be 21/24 months = 0.875. For the other trial durations, this can be calculated in a similar way.

The next penalty applied addresses the potential effect of a locally administered therapy in the study eye on the control eye, due to contamination of the fellow eye with the active agent through the bloodstream. Although we do not expect this to happen, we apply a conservative penalty of 5% in all designs that randomize across both eyes of 1 patient (contamination rate = 0.05, so retained effect: 1 – 0.05 = 0.95). Logically, in design 1, where patients instead of individual eyes will be randomized, the control eyes have no chance of contamination, and the contamination rate is set to zero.

So, for example, when calculating the Δ for a 2-year clinical trial with a paired-eye design and a hypothetical mean $\sqrt{\text{DDAF}}$ slope of 0.10 mm/year, the Δ between the 2 eyes would be 0.10 × 0.80 (inhibitory treatment effect) × 0.875 (retained study duration) × (1 - 0.05 [contamination rate]) = 0.067 mm/year, so the average progression in the treated group is expected to be 0.067 mm/year less than in the control group. Assuming equal mean values at baseline, the mean $\sqrt{\text{DDAF}}$ value in the treated eye will be 2 × 0.067 = 0.13 mm less than in the control eye after 2 years of treatment.

### Sample Size Calculations

Finally, after applying the penalties and calculating the resulting effect sizes, the estimated sample size for each design could be calculated. For all designs, the standard type I and II error rates of α = 0.025 (1-sided testing) and β = 0.20 were applied. For design 1—the 1-eye design—no intereye correlation between study eye and control eye had to be taken into account in the sample size calculations. Therefore, to estimate the total number of patients for each clinical trial duration with design 1, the following formula was used:

$$n_{total}=2\times \left(\frac{{2\times SD}_{slope}^{2}\times {\left(z_{1–\alpha }+z_{1–\beta }\right)}^{2}}{\Delta^{2}}\right)$$

in which n_total_ resembles the total number of patients (both arms) required in the clinical trial, SD_slope_ reflects the SD of the slopes of $\sqrt{\text{DDAF}}$ for the corresponding clinical trial duration and Δ is the effect size calculated for each design as described above.

For the sample size calculations of design 2 and 3, an extra step had to be taken to correct for the intereye correlation between the study and control eyes. In these designs, instead of calculating ${\text{SD}}_{\text{diff}}$ from ${2\times \text{SD}}_{\text{slope}}^{2}$, we calculated a modified SD that incorporates the ICC between the left and right eye of each patient, reflecting the SD of the difference in slopes between the eyes, as follows:

$$SD_{diff}=\sqrt{2\times \left(1–ICC\right)\cdot SD_{slope}^{2}}$$

Using ${\text{SD}}_{\text{diff}}$, the total number of patients (pairs of eyes) required for each clinical trial duration with designs 2 and 3 was estimated by:

$$n_{pairs}=\left(\frac{{SD}_{diff}^{2}\times {\left(z_{1–\alpha }+z_{1–\beta }\right)}^{2}}{\Delta^{2}}\right)$$

These formulas have earlier been described for use in ophthalmic studies by Gauderman et al.^11^ The ${\text{SD}}_{\text{diff}}$ could have been estimated directly from the differences between the left and right eyes, but the formula with the ICC reflects nicely the effect of asymmetric intereye progression: the higher the asymmetry, the lower the ICC and the larger the ${\text{SD}}_{\text{diff}}$. Higher asymmetry requires a larger sample size in the paired-eye designs. If the ICC is zero and the penalties are ignored, we would need 50% of the patients from the 1-eye design for the paired-eye design, as each patient has 2 eyes. Correlation between the eyes, i.e., a positive ICC, reduces the required number of patients even further. Note that some additional patients may need to be added to compensate for potential dropout during a clinical trial.

### Adjusting the ICC for the Unilateral Treatment Effect

Next, we simulated the clinical trial designs using our natural history data to assess the reliability of our sample size calculations. For each design, we randomly chose a number of patients that corresponded with the required sample size from the natural history data. One eye of each patient was randomly chosen as the treated eye. The mean $\sqrt{\text{DDAF}}$ slope of the treated eye was adjusted to reflect the expected outcome under an 80% treatment effect applying all necessary corrections as described above. Next, a paired *t* test was performed to compare the mean $\sqrt{\text{DDAF}}$ slopes between treated and nontreated eyes. This process was repeated 100 times for each design. We calculated the proportion of simulations yielding statistically significant results (2-sided *P* < 0.05), the ICC of the eyes without treatment, and the ICC after simulated treatment, and we calculated per simulation the ratio of the ICC after treatment to the ICC without treatment. We did this for study durations ranging from 2 to 5 years, and selected 1 correction factor based on these ratios. This correction factor was then applied to adjust the untreated ICC for the weakened correlation due to the treatment effect in 1 of the eyes. For example, when the initial ICC was 0.7 and the factor was 0.6, we used 0.42 as the adjusted ICC for the sample size calculations. Finally, we calculated the required sample size using the adjusted ICC and repeated the simulation process to generate the proportion of simulations yielding statistically significant results to validate our approach.

## Results

In total, of the 421 patients in the Radboudumc Stargardt cohort, 164 patients met the inclusion criteria; 93 patients were female (56.7%). Median age of STGD1 onset was 25.5 years (interquartile range [IQR], 16–47.3), median age during the most recent measurement was 48 years (IQR, 30–61), and median disease duration at timepoint 0 was 12 years (IQR, 7–19). All patients were eligible for the analyses of designs 1 and 2. Ninety-eight patients (59.8%) had DDAF atrophy in both eyes at their first eligible visit and were therefore eligible for the analysis of design 3. The demographics and patient characteristics of the cohort can be found in Table 1.

**Table 1:** Demographics and Patient Characteristics of the Included Cohort, Including the Preinclusion Cohort

Cohort	Number of Patients with ≥2 *ABCA4* Mutations	% Female	Median (IQR) Age of Onset (yrs)∗	Median (IQR) Age of the Most Recent Measurement	Mean Most Recent $\sqrt{\text{DDAF}}$ OD	Mean Most Recent $\sqrt{\text{DDAF}}$ OS
Preinclusion cohort	421	54	19 (11–40)	NA†	NA†	NA†
One-eye and paired-eye design	164	57	26 (16–47)	48 (30–61)	1.95	1.73
Paired-eye design with inclusion criteria	98	52	20 (15–44)	49 (31–64)	3.04	2.78

DDAF = definitely decreased autofluorescence; IQR = interquartile range; NA = not available; OD = right eye; OS = left eye.

∗ Age of onset was unknown for 35 patients in the preinclusion cohort, 8 patients included in the 1-eye and paired-eye design, and 4 patients included in the paired-eye design with inclusion criteria.

† Fundus autofluorescence images of not included patients were not graded, so no measurement results are available.

In the total cohort, the mean slope of $\sqrt{\text{DDAF}}$ progression was 0.134 mm/year, whereas in the patients with DDAF atrophy in both eyes at first measurement the progression was 0.188 mm/year. The SDs of the mean $\sqrt{\text{DDAF}}$ progression for every modeled clinical trial duration ranged from 0.171 to 0.323 mm/year and can be found in Table S2 (available at www.ophthalmologyscience.org).

In the paired design without inclusion criteria, differences in mean $\sqrt{\text{DDAF}}$ slope between the right and left eyes were skewed toward a faster mean progression of DDAF in the right eye for every clinical trial duration, ranging from 0.008 to 0.065 mm/year faster. In design 3, where both eyes should have DDAF atrophy at inclusion, no skewness was noticeable; the difference in mean DDAF slope ranged from –0.014 to 0.012 in favor of the right eye. Assumptions for the values of the sample size calculations for each design, clinical trial duration, and the assumed differences in growth rate between the right and left eyes (mean $\sqrt{\text{DDAF}}$ diff), were based on the descriptive statistics in Table S2, and can be found in Table 3, together with the ICC and the Δ per design.

**Table 3:** Values for SD $\sqrt{\text{DDAF}}$ Slope, ICC, and Δ per Trial Design

Trial Design	DDAF in both Eyes at Start	Trial Duration (yrs)	SD $\sqrt{\text{DDAF}}$ Slope (mm/yr)	Δ (mm/yr)	Uncorrected ICC
One-eye design	No	2	0.207	0.089	NA
		3	0.185	0.093	NA
		4	0.180	0.095	NA
		5	0.171	0.096	NA
Paired-eye design	No	2	0.207	0.089	0.400
		3	0.185	0.093	0.646
		4	0.180	0.095	0.709
		5	0.171	0.096	0.734
Paired-eye design with inclusion criteria	Yes	2	0.225	0.125	0.446
		3	0.199	0.131	0.640
		4	0.197	0.134	0.726
		5	0.183	0.136	0.754

DDAF = definitely decreased autofluorescence; ICC = intraclass correlation coefficient; SD = standard deviation.

### Simulations and Sample Size Calculations

Our simulations showed that the ICC changed during the clinical trials due to the unilateral treatment effect. The mean ratio of the mean simulated ICC at baseline to the mean simulated ICC after treatment was 0.62. Based on this, we applied a correction factor of 0.60 to the ICC. The ICC values before and after treatment, along with the proportion of significant simulations, are provided in Table S4 (available at www.ophthalmologyscience.org).

**Figure 1:**
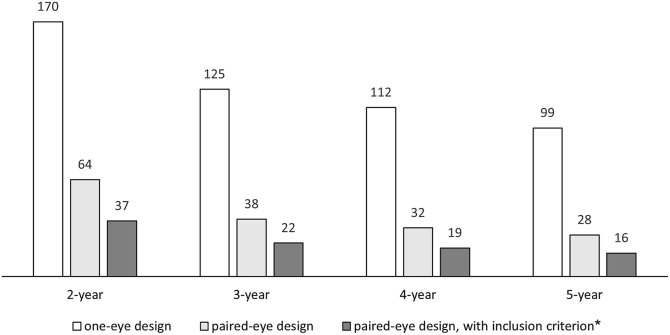
Total number of patients needed for each modeled clinical trial design: 1-eye design, paired-eye design, and paired-eye design with additional inclusion criterion. The number of patients needed is depicted for a 2-year to 5-year clinical trial duration. We assumed an inhibitory treatment effect of 0.8, a delay of 3 months, and in the paired-eye design, a contamination of 0.05. ∗Inclusion criterion: both eyes of the patient should have DDAF atrophy at first visit. DDAF = definitely decreased autofluorescence.

The sample sizes that are needed to reach sufficient statistical power vary greatly between the clinical trial designs. For the 1-eye design, when randomizing on patients, 170 patients are needed for a clinical trial with a duration of 2 years, decreasing to 99 patients in a 5-year clinical trial. When randomizing on the eyes of a patient, these numbers decrease to 64 and 28 patients for 2- and 5-year trial duration. When adding the inclusion criterion that both eyes should have DDAF atrophy at inclusion, the number of patients needed is further decreased to 37 and 16 patients for 2 and 5 years, respectively. The required numbers of patients for all clinical trial durations are shown in Figure 1.

## Discussion

This study shows that for a clinical trial in STGD1, with DDAF atrophy growth rate as the primary end point, a paired-eye design results in a much lower number of required patients to reach sufficient statistical power than a 1-eye design. This is of utmost importance for the emerging clinical trials in not only STGD1 but in all IRDs, and even in all ophthalmic diseases for which local treatment options are being developed.

The growth rate observed in our data set aligns with those reported in other studies when expressed in the same units. Shen et al^12^ reported a rate of 0.184 mm/year (radius root transformed) in a large meta-analysis, whereas Bassil et al^13^ found a rate of 0.20 mm/year. Our estimates ranged from 0.171 to 0.323 mm/year across different inclusion criteria based on different trial designs, confirming consistency with the existing literature.

Asymmetry between the 2 eyes of the same individual was consistently smaller than the variability between eyes of different individuals. This supports the efficiency of a paired-eye design, where the untreated fellow eye serves as a within-subject control. Additionally, a 1-eye design, being less efficient, requires approximately twice as many participants to achieve the same number of included eyes in the trial.

For a 5-year trial using a paired-eye design, approximately 28 participants would be sufficient to detect a treatment effect with adequate power. In contrast, for a 2-year clinical trial only 64 patients were needed when using a paired-eye design (assuming an ICC of 0.240), compared with 170 patients in a 1-eye design. In the field of IRDs, conducting a clinical trial with 170 patients would not be feasible, due to the rareness of these diseases. On top of that, a major part of the therapies that are being developed at the moment are mutation-specific,^14^, ^15^, ^16^, ^17^, ^18^ and patients need to be in a phase of disease in which they show disease progression,^19^ narrowing the eligible population for trials even more. Another factor decreasing the number of eligible patients for clinical trials is previous participation in a trial with a treatment with an irreversible effect, such as a gene therapy. Only when reducing the required sample size to a low 10-fold or even single digits can clinical trials be conducted in IRDs. The duration of a trial depends on the availability of eligible patients, the possibility to involve multiple clinical centers, and financial considerations, including both the costs and potential benefits associated with obtaining faster results.

However, despite their benefits, paired-eye designs are not often used. In 1992, Gauderman et al^11^ stated that a 2 eyes-per-subject design, in which 1 eye will receive experimental treatment and the fellow eye will receive control treatment, would be very powerful when there is reasonable correlation between the 2 eyes in the outcome measure. Nevertheless, to date the most used clinical trial design in ophthalmic randomized controlled trials is still the 1-eye design. Lee et al^5^ found in 2012 that of 69 randomized controlled trials in 4 major ophthalmic journals, only 13% used a paired-eye design and 19% another 2-eye design (for example, both eyes in the treatment group or both eyes in different treatment groups); 48% of the trials used a 1-eye design.^5^ Ten years later, Dong et al^4^ showed that not much had changed. Of 96 randomized controlled trials published between January 2020 and December 2021, only 10% used a paired-eye design, 22% a 2-eye design, and 52% a 1-eye design.^4^

The Food and Drug Administration (FDA) of the United States recommended against using a paired-eye design in its 2020 “Human Gene Therapy for Retinal Disorders | Guidance for Industry” publication. The FDA stated that the treated eye and contralateral eye may be in different disease stages, and that on top of that the disease progression may differ between these eyes during the clinical trial period.^20^

Unintended inclusion of patients with asymmetric deterioration is a risk for paired-eye designs. Especially with small sample sizes, the consequences of higher-than-expected asymmetry may have a severe negative effect on the power. To prevent this, inclusion criteria must be set. For the third design that we explored in our study, we set as an inclusion criterion that it must be possible to detect the progression of the end point in both eyes (so in our case, DDAF must be measurable in both eyes). In our study, this measure already caused a drop in the required sample size, but more stringent inclusion criteria can be set, including but not limited to requiring a similar progression of the end point in both eyes, securing a relatively high intereye correlation.

Additionally, the FDA mentioned that the different procedures in both eyes (treatment versus sham) may lead to unmasking, which will confound the study results. Although unmasking is a serious risk in paired-eye designs with functional outcomes, we do not expect accidental unmasking to influence the atrophy growth rate, a structural outcome, in a patient’s eye. This is because the grader of the FAF images, ideally from an independent reading center, will remain masked to the treatment. Another limitation of a paired-eye design is the impossibility of including patient-reported outcome measures, as 1 eye of the patient is treated and 1 eye serves as control.^21^

Although it may be very advantageous to use a paired-eye design, there are indeed limitations to it, as there are to every clinical trial design. Our study showed that for STGD1, disease progression was not very symmetric in both eyes of a patient, with an ICC of 0.40 for 2-year follow-up. However, even with a worst-case scenario of an ICC of 0.00, it is more efficient to evaluate a treatment in a paired-eye design trial than in a 1-eye design trial, because the number of available eyes is doubled in a paired-eye design.

In summary, this study demonstrates that adopting a paired-eye design in ophthalmic clinical trials, particularly for IRDs such as STGD1, offers large benefits over a 1-eye design. Disadvantages of the paired-eye design can be counteracted by issuing penalties in the sample size calculation. This approach reduces the number of patients required to conduct a trial, enhancing feasibility for trials in IRDs where patient availability is limited. Additionally, it lowers the associated trial costs.
