# Supplementary material for: A Comparison of Randomizing Either One Eye or Both Eyes in Clinical Trials for Stargardt Disease Type 1
Source: Ophthalmol Sci. 2025 Nov 26;6(2):101021. doi: 10.1016/j.xops.2025.101021 (PMC12818204; doi:10.1016/j.xops.2025.101021)
Supplement: Table S2 [file mmc1.pdf]

Table S2 All values used in the sample size calculations

Trial design	DDAF in both eyes at start	Trial duration (years)	Retained study duration	contamination rate	treatment effect	z(1-α)	z(1-β)	Mean $\sqrt{\text{DDAF}}$ slope (mm/year)	SD $\sqrt{\text{DDAF}}$ slope (mm/year)	Δ (mm/year)	Mean $\sqrt{\text{DDAF}}$ diff (mm/year)†	ICC $\sqrt{\text{DDAF}}$ after correction	SD $\sqrt{\text{DDAF}}$ diff (mm/year)	# of patients needed‡
one-eye design	No	2	0.875	0.05	0.8	1.96	0.84	0.134	0.207	0.089	NA	NA	NA	170
		3	0.916	0.05	0.8	1.96	0.84	0.134	0.185	0.093	NA	NA	NA	125
		4	0.938	0.05	0.8	1.96	0.84	0.134	0.180	0.095	NA	NA	NA	112
		5	0.950	0.05	0.8	1.96	0.84	0.134	0.171	0.096	NA	NA	NA	99
paired-eye design	No	2	0.875	0.05	0.8	1.96	0.84	0.134	0.207	0.089	0.038	0.240	0.255	64
		3	0.916	0.05	0.8	1.96	0.84	0.134	0.185	0.093	0.008	0.387	0.205	38
		4	0.938	0.05	0.8	1.96	0.84	0.134	0.180	0.095	0.012	0.425	0.193	32
		5	0.950	0.05	0.8	1.96	0.84	0.134	0.171	0.096	0.013	0.441	0.181	28
paired-eye design with inclusion criteria	Yes	2	0.875	0.05	0.8	1.96	0.84	0.188	0.225	0.125	0.012	0.268	0.272	37
		3	0.916	0.05	0.8	1.96	0.84	0.188	0.199	0.131	-0.014	0.384	0.221	22
		4	0.938	0.05	0.8	1.96	0.84	0.188	0.197	0.134	-0.002	0.436	0.209	19
		5	0.950	0.05	0.8	1.96	0.84	0.188	0.183	0.136	0.002	0.453	0.192	16

† OD minus OS

‡ α = 0.025, 80% power
