# Supplementary material for: A Comparison of Randomizing Either One Eye or Both Eyes in Clinical Trials for Stargardt Disease Type 1
Source: Ophthalmol Sci. 2025 Nov 26;6(2):101021. doi: 10.1016/j.xops.2025.101021 (PMC12818204; doi:10.1016/j.xops.2025.101021)
Supplement: Table S4 [file mmc2.pdf]

Table S4 Simulated values

Trial design	DDAF in both eyes at start	Trial duration (years)	Original ICC	Sample size without ICC correction	Proportion statistical significant (100 simulations)	Mean simulated ICC baseline	Mean simulated ICC after treatment	ICC ratio†	Corrected ICC‡	Sample size with corrected ICC	Proportion statistical significant (100 simulations)
paired-eye design	No	2	0.400	51	0.95	0.530	0.410	0.77	0.240	64	0.96
		3	0.646	22	0.83	0.664	0.430	0.65	0.387	38	1.00
		4	0.709	16	0.76	0.736	0.435	0.59	0.425	32	0.92
		5	0.734	13	0.70	0.764	0.441	0.58	0.441	28	0.94
paired-eye design with inclusion criteria	Yes	2	0.446	28	0.86	0.581	0.421	0.72	0.268	37	0.94
		3	0.640	13	0.75	0.677	0.400	0.59	0.384	22	0.96
		4	0.726	9	0.64	0.717	0.391	0.55	0.436	19	0.93
		5	0.754	7	0.69	0.761	0.365	0.48	0.453	16	0.87

† ICC ratio is the mean ratio of ICC after treatment to ICC without treatment after 100 simulations of each trial design

‡ Mean ICC ratio was 0.62. ICC correction factor was set at 0.60
